# Statistical Evaluation of Radiofrequency Exposure during Magnetic Resonant Imaging: Application of Whole-Body Individual Human Model and Body Motion in the Coil

**DOI:** 10.3390/ijerph16061069

**Published:** 2019-03-25

**Authors:** Wenli Liu, Hongkai Wang, Pu Zhang, Chengwei Li, Jie Sun, Zhaofeng Chen, Shengkui Xing, Ping Liang, Tongning Wu

**Affiliations:** 1 Division of Medical and Biological Measurement, National Institute of Metrology, Beijing 100029, China; zhangpu@nim.ac.cn (P.Z.); lichw@nim.ac.cn (C.L.); sunj@nim.ac.cn (J.S.); 2 School of Biomedical Engineering, Dalian University of Technology, Dalian 116024, China; wang.hongkai@dlut.edu.cn (H.W.); zhaofeng.chen@foxmail.com (Z.C.); 3 China Academy of Information and Communications Technology, Beijing 100191, China; xingshengkui@163.com; 4 XGY Medical Equipment Co. Ltd., Ningbo 315400, China; clarke_ic@163.com; 5 Ningbo Tongde Medical Equipment Technology Co. Ltd., Ningbo 315000, China

**Keywords:** numerical simulation, transmit coil, deformable human model, stochastic dosimetry, specific absorption rate

## Abstract

The accurate estimation of patient’s exposure to the radiofrequency (RF) electromagnetic field of magnetic resonance imaging (MRI) significantly depends on a precise individual anatomical model. In the study, we investigated the applicability of an efficient whole-body individual modelling method for the assessment of MRI RF exposure. The individual modelling method included a deformable human model and tissue simplification techniques. Besides its remarkable efficiency, this approach utilized only a low specific absorption rate (SAR) sequence or even no MRI scan to generate the whole-body individual model. Therefore, it substantially reduced the risk of RF exposure. The dosimetric difference of the individual modelling method was evaluated using the manually segmented human models. In addition, stochastic dosimetry using a surrogate model by polynomial chaos presented SAR variability due to body misalignment and tilt in the coil, which were frequently occurred in the practical scan. In conclusion, the dosimetric equivalence of the individual models was validated by both deterministic and stochastic dosimetry. The proposed individual modelling method allowed the physicians to quantify the patient-specific SAR while the statistical results enabled them to comprehensively weigh over the exposure risk and get the benefit of imaging enhancement by using the high-intensity scanners or the high-SAR sequences.

## 1. Introduction

Magnetic resonance imaging (MRI) is a widely used and powerful imaging technique for non-invasive clinical diagnosis [1]. In order to excite the detectable MR signals, its scanner typically includes a transmit (Tx) coil to generate a homogeneous circular polarized B_1_ field [2]. The induced eddy current is the primary radiofrequency (RF) absorption mechanism of the B_1_ field exposure, which will result in tissue heating. As a consequence, RF electromagnetic field (EMF) power deposition and the resulting tissue heating should be carefully managed, which becomes an important safety concern in sequence and coil design. Specific absorption rate (SAR) has been used to prevent excessive exposure [3]. International Electrotechnical Commission [4] defined the SAR averaged over whole body (wbSAR), over the head (hdSAR), over the partial body, and the peak SAR averaging over 10 g tissue (pSAR10g) as the limits.

For MRI exposure, overestimation for SAR may improperly restrict clinical MRI scans with new sequences, whereas underestimation of SAR can lead to tissue heating and thermal injury. Accurate SAR predication is critical for ensuring regulatory compliance, while completely exploiting the potential of the MRI system because of optimizing the imaging quality by repetitive scans or increasing the emissions in sequence resulted in higher SAR [5].

At present, SAR is usually non-invasively characterized using numerical simulations. A representation of the patient-specific SAR depends on the coil model and the individual model [6]. To date, accurate individual models are usually reconstructed using MRI [7]. In this case, the high-intensity scanner and high-SAR sequence were frequently applied to present the precise anatomical details. Non-rigid registration using the brain [6] and breast model libraries [8], tissue simplification [9,10], or body truncation [5] techniques allowed generating an individual human model by a low-intensity scan with an SAR-reduced sequence [6,11]. 

Nevertheless, the abovementioned methods either focused on partial body modelling or obscured the anatomical and physiological details which may influence the SAR. In this case, it is desirable to derive the a priori knowledge for the measurable anthropometric parameters (e.g., profile, height and weight) and the internal anatomy. However, the relationship was too complicated to be quantified [12]. 

Our recently developed deformable Chinese phantom [13,14] may help resolve the dilemma. In these studies, deformable human torso and head phantoms were constructed by learning the inter-subject anatomical variation from a segmented computed tomography (CT) dataset of healthy Chinese adults. To match the personal anatomy, the deformable phantoms were registered to individual body surface, which can be obtained by a 3-D surface optical scan. As a consequence, the individual model can be generated in less than 10 minutes using a personal computer and it is free of an MRI scan. The applicability of this kind of model in patient-specific SAR assessment could be promising but the resulting errors should be investigated, and some further work needs to be conducted to finalize the whole-body modelling (e.g., the limbs cannot be generated by the method because the CT dataset did not include this part).

Positioning of the patient in the scanner usually involves random misalignment, which is inevitable in clinical scans [15]. Among the three directions, the shift along the long-axis of the coil (Z-axis) was roughly controlled compared to the other directions which were practically confined by padding or spacers. The Z-axis alignment was based on several body landmarks (e.g., shoulder, groin) and the shift could introduce substantial SAR change [16]. Besides, the body tilt was frequently observed due to the inaccuracy in coil installation (leading to a relative rotation between the excitation port and the body) or the slight motion of the patient. The extent of variation has been evaluated for the Z-axis shift mainly using the deterministic method [5,16] whilst no report was available for the body tilt. In comparison, the stochastic approach addressed the SAR variability based on the probability distribution of the input variables [12,17,18,19,20]. The statistical results would be appropriate for MRI exposure assessment because clinical physicians could manage SAR at different risk levels when weighing over the benefit from the SAR-enhanced sequence.

In this work, we proposed a whole-body individual modelling method, including deformable phantom and tissue simplification, to evaluate the patient-specific exposure to B_1_ field of MRI. For this purpose, a commercial Tx coil was numerically reconstructed and verified by measurement. The whole-body individual model could be efficiently generated with high accuracy and contained 37 (male) and 34 (female) different tissues. Stochastic dosimetry was conducted using a surrogate model [21] constructed by polynomial chaos [22]. The exposure variability due to the Z-axis misalignment and tilt of the subject was quantified. Both the deterministic and stochastic results validated the application of the individual modelling technique in evaluating the RF exposure of Tx coil. The statistical results fitted well for the need for exposure risk management in MRIs. The procedures as well as the modelling method can be used for efficient patient-specific SAR during MRI assessment. 

## 2. Materials and Methods 

### 2.1. Whole-Body Individual Modelling

The deformable human phantoms were recently generated for the human torso [13] and head [14] while representing the physical parameters in a given Chinese population. Statistical shape model [23] was used to learn about the inter-subject anatomical variation from a CT dataset containing 79 healthy Chinese, whose tissues were either automatically segmented or mapped using the anatomical templates [24], depending on the specific imaging contrast. To match the personal anatomy for individualized modelling, the deformable phantoms were registered to the individual body profile (can be achieved by an optical scanner) using an active shape model [25]. This was an efficient method (less than 10-min’s calculation on a personal computer) free of MRI scans. However, the abovementioned registration method cannot be applied for limbs because they were not included in the CT database. In the experiments, we assumed that the limbs were homogeneous with muscle. 

**Figure 1:**
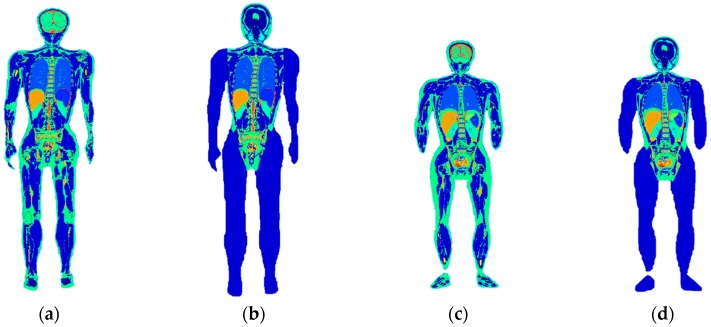
Human models used in the study: (**a**) Chinese adult male model by manual segmentation; (**b**) individualized adult male model; (**c**) Chinese adult female model by manual segmentation; (**d**) individualized adult female model.

In order to validate the proposed modelling method, we selected the Chinese adult female and male models [26] as references. The models were manually segmented and reconstructed from cryo-section slices by 10 anatomical experts at the resolution of 1 mm^3^. Rigorous quality control has been ensured in the process [27]. They were assumed to present the individual anatomical structure with high confidence. Their profiles were extracted and used for whole-body individual modelling with the procedures described in the previous paragraph. The human models reconstructed by manual segmentation and by the approach as we introduced in this study are shown in Figure 1.

The tissues in the abovementioned models are listed in Table 1. The number of the tissues for the male models was 66 (manually segmented model) and 37 (individualized model). As for the female models, the manually segmented model contained 62 tissues, while the individualized model had 34 tissues. 

### 2.2. Computational Method

#### 2.2.1. Deterministic Simulations

Finite-Difference Time-Domain (FDTD) is commonly used for electromagnetic dosimetry [28]. It works in time domain and is capable of solving the time response of a pulse for heterogeneous tissues. In contrast, the method has difficulty to handle the large-scale problem with a fine resolution, which introduces huge memory cost. In addition, the steady-state solutions are obtained by simulating the whole transient time response of the system and this can result in very long simulation times for strong resonators. To overcome the drawbacks, we used the equivalent source [29] and the adaptive voxels [28] to reduce the simulation volume. 

**Figure 2:**
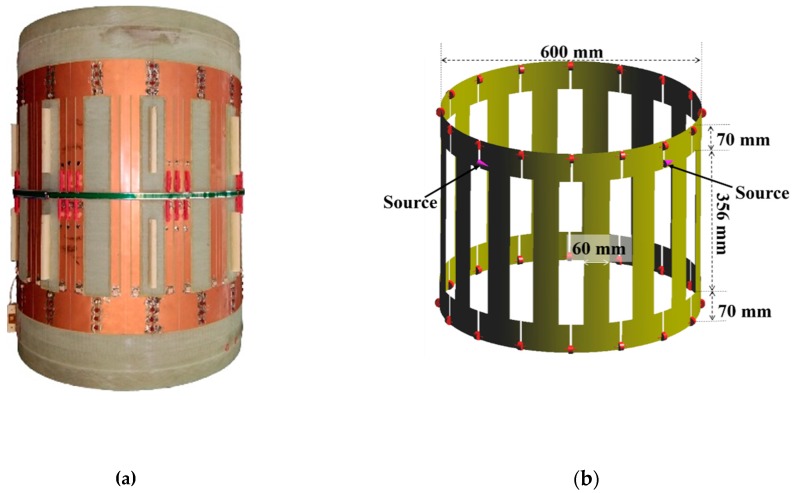
Structure of the MRI Tx coil: (**a**) Tx coil used in the experiment; (**b**) CAD model of the coil, the red dot indicates the positions of the capacitors.

Taking the coil used in the experiments for an example, a generic 1.5 T 16-rung Tx coil fabricated by XGY Medical Equipment Co. Ltd, (Ningbo, Zhejiang, China) was used in the study (Figure 2a). The dimensions of the coil are shown in Figure 2b. The coil model was tuned to 64 MHz by adjusting the between-rung capacitors. By the method, the calculated return loss (S_11_) was −12.7 dB, close to the measured one (−13.3 dB). 

The incident field was obtained for this Tx coil model which was discretized with an adaptive grid schema (496 × 496 × 411 = 101.52 MCells). The result was used as input for the Huygens Box simulations [28] with the anatomical model at a uniform grid resolution of 2 mm^3^.

All simulations have been performed with SEMCAD V17.2.1 (SPEAG, Zurich, Switzerland). For the approximate 36.8 MVoxel of the entire simulation space for the human model, the calculation time was around 0.5 h per simulation on a hardware accelerated cluster (CPU: 2 × Xeon E5-2630, 2.2 GHz; Memory on board: 256 GB; GPU: 2 × NVIDIA Tesla K40c with 24 GB memory in total). Two quadrature feeding ports were located in the upper-end ring. The numerical simulations were performed in a multi-port simulation mode with E-field data superimposed from the results of the two quadrature feeds. Frequency-dependent dielectric properties were adopted from the databases of Gabriel et al. [30] and Hasgall et al. [31]. The values were directly used for the manually segmented models. In contrast, we need to homogenize the dielectric properties of the tissues for the individual models as shown in Table 1. The homogenization was based on the mass as shown in (1):

(1) $${\tilde{\varepsilon }}_{\mathrm{homogenized}}=\frac{\sum_{i=1}^{N}{\tilde{\varepsilon }}_{i}m_{i}}{\sum_{i=1}^{N}m_{i}}=\sigma +j\omega \varepsilon \prime$$

where, ${\tilde{\varepsilon }}_{\mathrm{homogenized}}$ is the complex dielectric properties of the homogenized tissue; ${\tilde{\varepsilon }}_{i}$ and $m_{i}$ denote the complex dielectric properties and mass of the ith tissue contained in the homogenized tissue; σ is the conductivity; and ε′ is the relative permittivity. 

The homogenized results are shown in Table 2.

**Figure 3:**
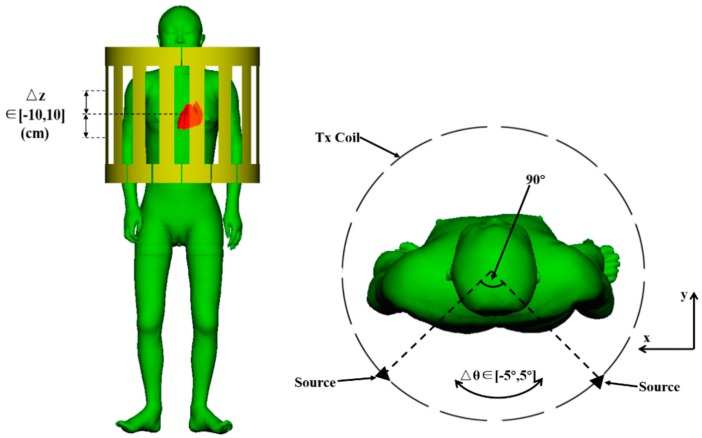
Positioning of the human models in the coil.

The human models were positioned in the coil with their long axis aligning with the Z-axis of the coil. The middle level of the coil along the Z-axis was aligned to the central heart level of the human models. This was defined as the standard position for the human model in the coil. To evaluate the variability due to the body motion in the coil, the models can move along the Z-axis for ±10 cm and rotate for ±5°. The configurations are shown in Figure 3. The minimum distance between the arms and the coil was 7 cm for the adult female model and 6 cm for the adult male model.

#### 2.2.2. Stochastic Dosimetry on Z-Axis Shift and Body Tilt

Stochastic methods are capable of evaluating the exposure in realistic conditions [12]. The system output (i.e., the dosimetric quantity under assessment) is acquired on the basis of a few observations of the system output obtained by deterministic simulations. Recently, spectral method, in particular, polynomial chaos (PC), has been widely used in the variability analysis of bioelectromagnetic dosimetry at a lower computational cost [32]. It approximates the system outputs by a surrogate model using a series of orthogonal polynomial basis (ψ(X)) as in (2).

(2) $$SAR=M(X)=\sum_{0}^{P-1}a_{j}\psi_{j}(X)+e$$

where *X* is the random input vectors made of the variables under assessment (Z-axis shift and body tilt in our case), $\psi_{j}(X)$ are the polynomials from ψ(X), $a_{j}$ are the coefficients to be estimated, and *e* is the truncation error. *P* is the size of the polynomial basis and is calculated as (3):

(3) $$P=\left(\begin{array}{c} K+p\\ p \end{array}\right){=C}_{p+K}^{p}$$

where *p* is the maximum order of ψ(X) and *K* is the number of input variables. If the input variables are independent, *ψ*(*X*) can be represented as (4):

(4) $$\psi \left(X\right)=\prod_{j=1}^{K}\pi_{\alpha_{j}}(x_{j})=\pi_{\alpha_{1}}(x_{1})\times \ldots ..\times \pi_{\alpha_{K}}(x_{K})$$

where $\pi_{\alpha_{j}}$ is a family of polynomial orthogonals with respect to the probability density function (PDF) of each input variable $x_{i}$, and $\alpha_{j,1\leq j\leq K}$ represents the maximum degree of the polynomials in $\pi_{\alpha_{j}}$ [22,33]. Practically, the probability density of the Z-axis shift and rotation were assumed to follow Gaussian distribution, respectively. Therefore, Hermite polynomials were selected. 

Usually, Least Angle Regression (LAR) algorithm [34] is used to estimate the coefficient of PC. This algorithm is based on least-square regression with respect to the series of observations of the system output $SAR_{o}=\left\{sar_{1}^{},sar_{2}^{},\ldots ,sar_{N}^{}\right\}$. The validation of the surrogate model was based on a leave-one-out cross validation approach (LOOCV) [35]. This technique can reduce the size of the experimental design. By this way, the observation set, obtained with deterministic dosimetry from the experimental design to an experimental design $X_{o}=\left\{x_{1}^{},x_{2}^{},\ldots ,x_{N}^{}\right\}$, is recursively divided into two subsets, the learning set to build the surrogate model and the testing set to calculate the resultant errors. The best PC expansion among the ones generated by LAR is then chosen through LOOCVs, using the corrected relative leave-one-out error ($\varepsilon_{\mathrm{LOO}}$ [36]. $\varepsilon_{\mathrm{LOO}}$ is based on the estimation of the mean square error of the model as shown in (5)

(5) $$\varepsilon_{\mathrm{LOO}}=\frac{1}{N}\sum_{i=1}^{N}{\left({\hat{M}}_{-J}(x_{i})-M(x_{i})\right)}^{2}$$

where, ${\hat{M}}_{-J}$ is the model based on N-1 simulated SAR values, $\left\{sar_{1}^{},sar_{2}^{},\ldots ,sar_{N}^{}\right\}-\left\{sar_{J}^{}\right\}$.

The PC procedure should be repeated by increasing the size N of the experimental design and changing the maximum degree p of the polynomials ψ(X) until the achieved error is below a given threshold $1-Q^{2}=\frac{\varepsilon_{LOO}}{\sigma_{sar}^{2}}$, where $\sigma_{sar}^{2}$ is the variance of the output SAR values.

We used Latin Hypercube Sampling (LHS, [37]) to generate the input variable vectors. The LHS technique is a multidimensional version of the stratified sampling method. It has the advantage of generating a set of samples that more precisely reflect and explore all the distributions with substantially fewer samples.

### 2.3. E-Field Measurement in the Empty Coil

**Figure 4:**
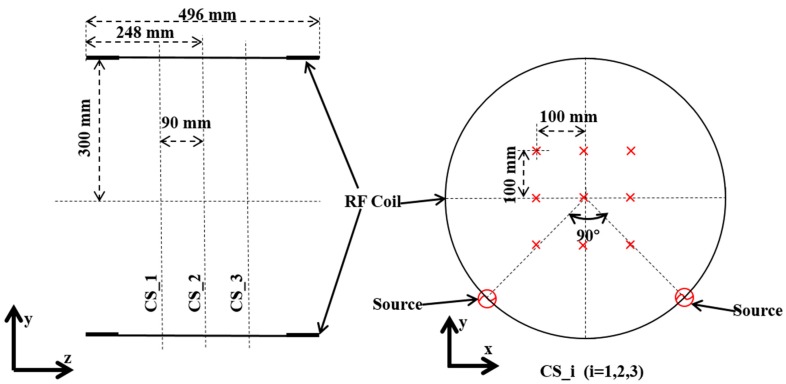
Field measurement for the Tx coil. CS_i (i = 1, 2, 3) indicates the three measurement surfaces in the coil, which were separated by 90 mm. “x” indicates the position of the measurement point.

To confirm the field distribution by numerical modelling of the coil, we measured the E-field strength on 27 points distributing on three cross-sections perpendicular to the Z-axis of the coil using E-field probe (ER3DV6) and H-field probe (H3DV7) with Easy4MRI system (SPEAG, Zurich, Switzerland). The measurement configuration is shown in Figure 4. We performed the E-field measurement with the sequence of the modulated rectangular pulse for comparison. The validation measurement and simulation were conducted in the empty coil.

## 3. Results

### 3.1. Whole-Body Individual Models

We compared the total mass of the different models as well as some major tissues/organs in the human models in Table 3. Their registration accuracy is also compared in the table.

The deviation of mass for the entire body, skin, fat, muscle, bones, and brain were less than 10%. The deviation of mass for the major organs was generally less than 20% but the registration error (Dice) was rather large, ranging from 61% to 81%. 

### 3.2. Comparison for the Simulation and the Measurement Results in the Empty Coil

**Figure 5:**
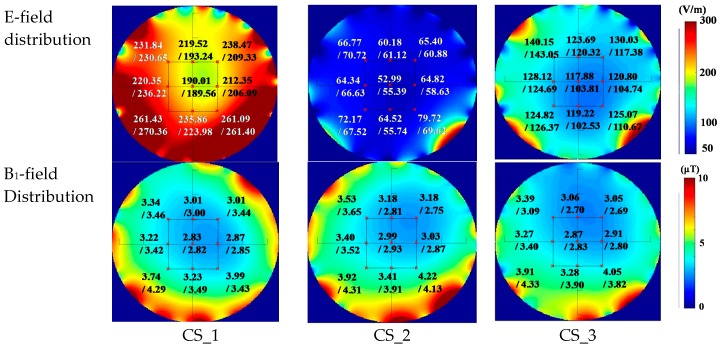
The simulated and the measured RMS field values on the three selected slices. The values were presented by “simulated result/ measured result”. The measured points were indicated by red x. The power to the coil was 6 kW (in measurement) and 3.5 kW (in simulation).

The calculated and the measured E and B_1_ field results are shown in Figure 5. 

The incident power was not identical for the simulation and measurement due to the inaccuracy in modelling. The discrepancy could be adjusted and compensated in simulations. In fact, the similarity of field distribution was a much more important factor. The mean deviation of the amplitude on these points was 6.13%, with a standard deviation of 3.92%. The maximum deviation was 14.42%. 

### 3.3. Deterministic Results

**Figure 6:**
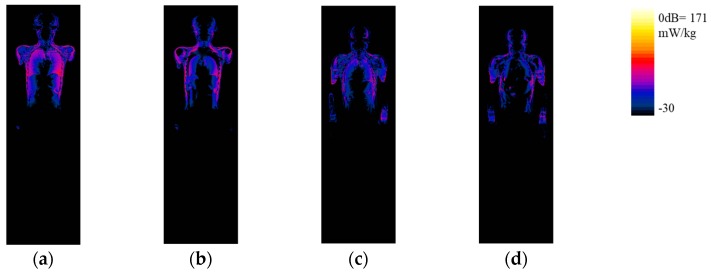

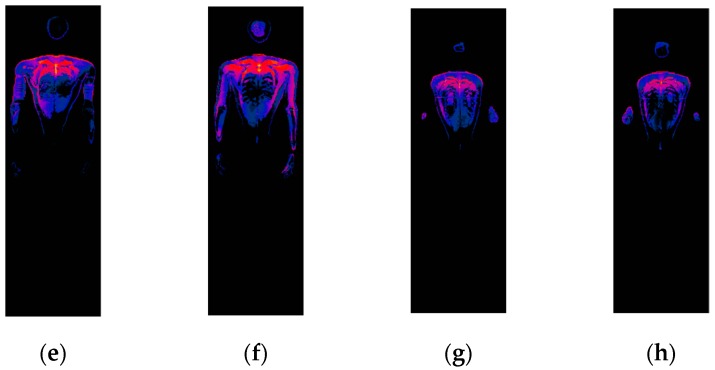
SAR results for the models at the standard position in the coil. (**a**–**d**) SAR distribution at the central coronal slice for the manually segmented Chinese adult male model, individual male model, manually segmented Chinese adult female model, and individual female model, respectively; (**e**,**f**) SAR distribution at the coronal slice which includes pSAR10g for the manually segmented Chinese adult male model, individual male model, manually segmented Chinese adult female model, and individual female model, respectively. The positions of pSAR10g are indicated in the figures. The results were normalized to a net incident power of 1 W.

The SAR distribution on the coronal slices is shown in Figure 6.

wbSAR and pSAR10g for the standard position are shown in Table 4.

The difference for wbSAR between the manually segmented models and the individual models was below 4% whilst the difference for pSAR10g between these models was less than 10%. The location of pSAR10g was very close between the manually segmented and the individual models.

### 3.4. Statistical Results

By LOOCV, a total of 100 experiments introduced the errors of less than 2% for the wbSAR results (1.12% for the Chinese adult male model, 0.2% for the individual male model, 0.5% for the Chinese adult female model, and 0.1% for individual female model). In comparison, the errors were less than 5% in terms of pSAR10g (4.2% for the Chinese adult male model, 1.2% for the individual male model, 4.8% for the Chinese adult female model, and 1.8% for the individual female model).

**Figure 7:**
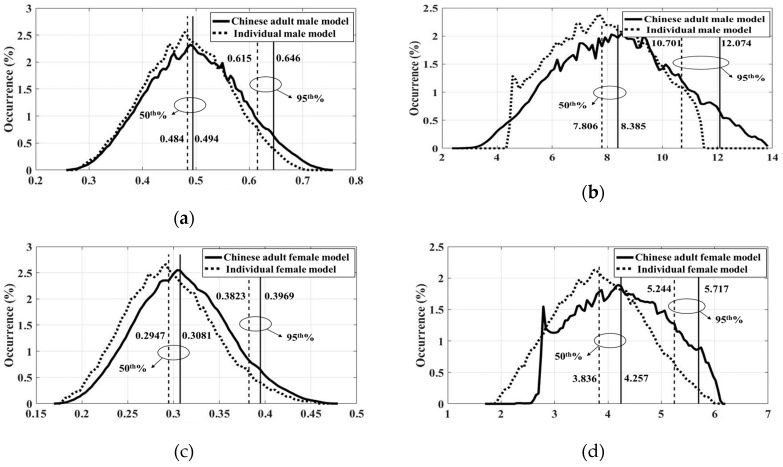
SAR occurrence calculated by surrogate model: (**a**) wbSAR for the male models; (**b**) pSAR10g for the male models; (**c**) wbSAR for the female models; and (**d**) pSAR10g for the female models.

Based on the obtained surrogate model, the distribution of wbSAR and pSAR10g due to body motion in the coil is plotted in Figure 7.

The results indicated that the individual models yielded similar SAR results even for various percentiles. For example, at the 50th percentile, the deviation for wbSAR from the male models is 2.07% and 4.54% for the female models. At the 95th percentile, the deviation is 5.04 % for the male models and 3.82% for the female models. Deviation for pSAR10g ranged from 7% to 13% at the 50th and 95th percentile.

## 4. Discussion

The models generated by the proposed modelling technique gave satisfactory accuracy of SAR estimation compared to the available fast individual modelling methods which demonstrated a deviation of 10–30% for pSAR10g [6,9]. The advantage of accurate SAR representation could be attributed to the anatomical details preserved by the proposed modelling technique. As known, the tissue distribution is essential to the local SAR. In total, more than 30 different tissues/organs were included in the individual model, which thus benefited for an accurate local SAR estimation. Another advantage of the proposed methodology is also obvious: The variables could be eventually traced to several anthropometry parameters (e.g., height and weight), which were dosimetrically important. This is a prospective method for individual modelling even without an optical or low-SAR MRI scan.

The proposed modelling technique learned the inter-subject anatomical variation from the medical image dataset consisting of 79 healthy Chinese individuals (41 males and 38 females). The representation of the database could be enhanced by the inclusion of more subjects. To note, since the CT dataset was from the healthy subjects, the modelling method cannot be directly applied to the subjects with internal abnormalities, e.g., tumors. Another point that underpinned the modelling technique was the representation of the anatomical templates used for the registration of the low-contrast organs. The current templates were created mainly for demonstrative visualization so that the shapes of the organs were stylized with their profiles being significantly smoothed. Although we have achieved good SAR accuracy, the organ’s registration accuracy as well as the SAR results could be potentially improved by choosing the confirmed anatomically correct templates. To note, including the individual information from other anatomical datasets, even for partial body, can help refine the deformable models. For example, Li et al. [8] provided a feasible approach to fuse the individual breast to the whole-body model. 

The limbs were simplified with homogeneous muscle due to the lack of the data for this part. Admittedly, change for the tissue composition can modify the wave propagation inside the body. Muscle was selected as the tissue for the limbs because it had a higher conductivity and permittivity compared to fat. Hence, it may indicate that very significant RF power absorption and conservative results were expected. By numerical comparison, the errors for wbSAR, pSAR10g and the locations were usually less than 10%, which was superior to the existing individual modeling methods [6,9]. Moreover, we analyzed the application of the simplification method to the limbs of the Virtual Family models [38]. Very similar results are presented as well (resulting pSAR10g deviation for less than 5% when the hands and the body were separated). The results were consistent with the previous studies indicating the simplification of the heterogeneous whole-body models using muscle–fat–lung tissues [9]. So, we concluded that limbs had much simpler tissue distribution and our simplification would not pose significant errors. Upper limbs have an important influence on the formation of RF loops when the hands contacted the wrist or hip. In practice, an electrical isolation has been recommended to insert between the arms and the torso as well as between the legs to avoid conducting loops [15,39]. Our study used the similar posture and prevented the first hotspots on the contacting parts. The optical scan can obtain the profile of the individual limbs. By this way, the real distance between the arms and the coils was kept. This case was very similar to cerebrospinal fluid (CSF). We cannot identify CSF from the CT dataset although its dielectric properties were very different compared to the other brain tissues. However, from Table 4, we found that the simplification resulted in less than 5% (in terms of errors of hdSAR) for the simulations.

The results demonstrated the SAR equivalence between the individual models and referenced models. Statistical results also demonstrated that the whole-body individual models could represent the SAR variation due to Z-axis misalignment and body tilt. Statistical results on Z-axis misalignment and the body tilt presented the SAR values for various percentiles. The reasonable safety margin rather than an overestimation factor can be determined upon SAR statistics. Setting the SAR limit to various percentiles covered different number of cases. The much more accurate SAR management can be performed and the physician can decide on the application of the novel sequence depending on the expectation of risk.

Two independent factors for body positioning in the coil were studied. Other sources also contributed to SAR variability including the ones from the dielectric properties and coil models. However, it was difficult to simultaneously take them into consideration due to the curse of dimensionality in high-dimensional problems [40]. The possible solutions may include sensitivity analysis and principle component analysis to determine the factors with dosimetric importance. In reality, the physical variables, such as body size and weight, influenced SAR as well. Researchers attempted to characterise the relationship so as to estimate the subject-specific exposure dose or to determine the conservative exposure dose for a population. For example, Murbach et al. discussed the relationship between the RF absorption and anatomy, but only using five human models [16]. Shao et al. statistically discussed the SAR variability due to the variation of the head volume using the unscented transform method [12]. In their work, the different head models were simply scaled from the head model of Duke [7], and the anatomical validity of the derived models was not addressed. By our method, the individual models were generated and, its resultant SAR difference was demonstrated to be less than 5% (wbSAR) and 10% (pSAR10g). So, we assumed that the individual models well-represented the reference models. Then, we focused our research on variability analysis for the body motion in the coil. In effect, the proposed modeling method can generate the human models representing some pre-defined physical and anatomical features, which is essential for statically evaluating the dosimetric results over a specific population. This is another important issue in MRI RF exposure assessment and we will discuss it in future studies.

The uncertainty for assessing the patient–subject SAR has multiple contributors in addition to human modelling. The model of the Tx coil and the numerical method are also important for accurate SAR prediction. In our experiments, we verified the simulated E-field strength in the empty coil using the measurement data from several points with the RMS value. The purpose was to confirm the Tx model rather than a rigorous uncertainty analysis. In general, the uncertainty for the measurement positioning was about 6–7%. According to the calibration documents, the uncertainty of the probes (with the experimental sequence) was 9.6% (k = 2). The uncertainty was around 50% for coil modelling [41,42]. Different numerical methods contributed approximately 6–15% of the uncertainty [43,44]. The information could be referred to for uncertainty estimation.

The deformable model was generated based on the dataset from the Chinese population. A similar method could be applied to other ethnicities when the relevant dataset is available. The body coil was a common commercial product without special optimization. The individual modelling did not use the tissue cluster based on the dielectric similarity [9], thus it is not frequency dependent. Therefore, the methodology as well as the results could be expected to be applied to the coils working at other Larmor frequencies. 

In the study, we removed the RF shield of the coil. The simplification was to facilitate the experiments for probe positioning as well as numerical modelling. The purpose of the study was to investigate the applicability of the human modelling method to the evaluation of the individual MRI RF exposure. The MRI coil model presented in the study acted as a source for exposure. Therefore, the detailed structure was not an influential factor on the conclusion. In practice, researchers can adopt any commercial coil in analysis. 

## 5. Conclusions

In this study, the efficient and low-SAR method to evaluate the patient-specific SAR during MRI scan was presented. We utilized a deformable atlas to render the individual trunk and head model based on the inter-subject anatomical variation learned from the CT dataset. The tissues in the upper and lower limbs were simplified to muscle. In that case, an optical scan or low-SAR MRI scan to obtain the individual profile was sufficient for an accurate patient–subject SAR evaluation compared with the current methods because a lot of individual anatomical and physical details were preserved. Since misalignment and body tilt were almost inevitable in the clinical scan, we statistically analyzed the dosimetric influence of the two factors. The results demonstrated that the proposed modelling method can yield similar statistical results compared with the reference models. SAR values for different percentiles were given using surrogate models reconstructed by polynomial chaos. The statistical results provided useful information when weighing over the exposure risk and the benefit of an SAR-enhancing coil/sequence.

## Figures and Tables

**Table 1:** Tissues for the different models.

Chinese Adult Male Model	Individualized Adult Male Model	Chinese Adult Female Model	Individualized Adult Female Model
Aqueous humor	Eyes	Aqueous humor	Eyes
Cornea		Cornea	
Cortex_of_lens		Cortex_of_lens	
Sclera		Sclera	
Retina		Retina	
Vitreous_body		Vitreous_body	
Lens_nucleus		Lens_nucleus	
Iris		Iris	
Lacrimal_apparatus		Lacrimal_apparatus	
Brain_stem	Brain	Brain_stem	Brain
Cerebral_dura_mater		Cerebral_dura_mater	
Cerebral_grey_matter		Cerebral_grey_matter	
Cerebral_white_matter		Cerebral_white_matter	
Hippocampus		Hippocampus	
Hypophysis		Hypophysis	
Hypothalamus		Hypothalamus	
Cerebellum		Cerebellum	
Cerebrospinal_fluid		Cerebrospinal_fluid	
Cartilage	Cartilage	Cartilage	Cartilage
Large_artery_wall		Large_artery_wall	
Large_vein_wall		Large_vein_wall	
Laryngeal_cartilages		Laryngeal_cartilages	
Nerve		Nerve	
Vestibulocochlear_nerve		Vestibulocochlear_nerve	
Diploe	Skull	Diploe	Skull
Teeth		Teeth	
Bile	—	Bile	—
Bladder	Bladder	Bladder	Bladder
Blood	Blood	Blood	Blood
Cholecyst	—	Cholecyst	—
Cortical_bone	Cortical_bone	Cortical_bone	Cortical_bone
Fat	Fat	Fat	Fat
Heart	Heart	Heart	Heart
Internal_ear	Internal_ear	Internal_ear	Internal_ear
Intervertebral_disc	Intervertebral_disc	Intervertebral_disc	Intervertebral_disc
Kidney	Kidney	Kidney	Kidney
Large_intestine	Intestines	Large_intestine	Intestines
Enteric_cavity		Enteric_cavity	
Ligament	Ligament	Ligament	Ligament
Liver	Liver	Liver	Liver
Lung	Lung	Lung	Lung
Lymph_node	Lymph_node	Lymph_node	Lymph_node
—	—	Mammary_gland	Mammary_gland
Intrinsic_laryngeal_muscle	Muscle	Intrinsic_laryngeal_muscle	Muscle
Muscle_belly		Muscle_belly	
Tongue		Tongue	
Muscle_tendon		Muscle_tendon	
Nucleus	Nucleus	Nucleus	Nucleus
Optical_nerve	Optical_nerve	Optical_nerve	Optical_nerve
Pancreas	Pancreas	Pancreas	Pancreas
Pineal_gland	Pineal_gland	Pineal_gland	Pineal_gland
Prostate	Prostate	—	—
Red_bone_marrow	Red_bone_marrow	Red_bone_marrow	Red_bone_marrow
Salivary_gland	Salivary_gland	Salivary_gland	Salivary_gland
Skin	Skin	Skin	Skin
Spinal_cord	Spinal_cord	Spinal_cord	Spinal_cord
Spinal_dura_mater	Spinal_dura_mater	Spinal_dura_mater	Spinal_dura_mater
Spleen	Spleen	Spleen	Spleen
Spongy_bone	Spongy_bone	Spongy_bone	Spongy_bone
Stomach	Stomach	Stomach	Stomach
Stomach lumen		Stomach lumen	
Testis	Testis	—	—
Thoracic_gland	Thoracic_gland	—	—
Thyroid	Thyroid	Thyroid	Thyroid
Trachea	Trachea	Trachea	Trachea
Ureter	Ureter	Ureter	Ureter
Vessel	Vessel	—	—

**Table 2:** Dielectric properties for the homogenized tissues of the individual models.

Homogenized Tissues	Conductivity (S/m)	Relative Permittivity
Eyes	1.24	70.96
Brain	0.69	72.35
Cartilage	0.60	66.76
skull	0.13	26.38
Muscle	0.69	72.24
Stomach	0.88	85.81

**Table 3:** Comparison for the major tissue/organs of the different models.

Tissues	Chinese Adult Male Model (kg)	Individual Male Model (kg)	Weight Deviation (%)	Dice ^1^ (%)	Chinese Adult Female Model (kg)	Individual Female Model (kg)	Weight Deviation (%)	Dice (%)
**Total weight**	63.26	66.70	5.44	/	53.47	54.99	2.84	/
**Fat**	21.54	23.32	8.26	/	17.10	18.81	10.00	/
**Muscle**	22.37	24.23	8.31	/	16.23	16.03	−1.20	/
**Skin**	3.79	3.77	−0.57	/	3.14	3.15	0.27	/
**Bones**	8.42	9.01	7.00	/	5.96	6.22	4.36	/
**Brain**	1.38	1.39	−1.49	81.08	1.30	1.26	−2.67	78.75
**Heart**	0.42	0.35	−17.00	69.49	0.27	0.30	11.11	69.98
**Kidney**	0.26	0.29	11.53	69.55	0.22	0.19	−13.63	63.83
**Liver**	2.05	1.84	−10.24	63.17	1.17	1.02	−12.80	61.76
**Lung**	1.02	0.90	−11.76	63.39	0.93	0.86	−7.76	68.87
**Spleen**	0.19	0.16	−15.79	61.18	0.18	0.14	−16.67	63.22
**Stomach**	0.76	0.74	−2.63	72.32	0.59	0.67	13.55	69.70

^1^ Dice (%) is calculated by $\frac{2\left|X\cap Y\right|}{\left|X\right|+\left|Y\right|}\times 100\%$, where X and Y are the voxels of the organ belonging to the manually segmented model and the individual model, respectively.

**Table 4:** Deterministic simulation results for the models at the standard position in the coil.

Human Models	wbSAR ^1^	Deviation (%) ^2^	hdSAR ^3^	Deviation (%)	pSAR10g	Deviation (%)	Location of pSAR10g
Manually segmented male model	0.48 mW/kg	−2.62	0.27 mW/kg	3.20	8.39 mW/kg	−7.55	(86, 85, 489)
Individual male model	0.47 mW/kg		0.28 mW/kg		7.76 mW/kg		(101, 86, 492)
Manually segmented female model	0.31 mW/kg	−3.06	0.21 mW/kg	4.53	4.24 mW/kg	−9.09	(93, 79, 461)
Individual female model	0.30 mW/kg		0.22 mW/kg		3.85 mW/kg		(96, 82, 463)

^1^ wbSAR is the SAR averaged over the entire body. ^2^ Deviation is calculated by the difference of the two models divided by the value from the manually segmented model. ^3^ hdSAR is the SAR averaged over the entire head. The results were normalized to net incident power of 1 W.
